# Supplementary material for: Quantifying emergent complexity
Source: Patterns (N Y). 2026 Jan 9;7(1):101472. doi: 10.1016/j.patter.2025.101472 (PMC12827727; doi:10.1016/j.patter.2025.101472)
Supplement: Document S1. Figures S1–S3 and Notes S1–S4 [file mmc1.pdf]

Patterns, Volume *7*

Supplemental information

Quantifying emergent complexity

Erik Hoel

Quantifying emergent complexity: Supplemental Information

Erik Hoel*[1]

[1]Allen Discovery Center, Tufts University, Medford, MA, USA

November 25, 2025

*erik.hoel@tufts.edu

Supplemental Notes

S1 Causal primitives (and their generalizations) are sensitive to noise and common causes.

Previous research on causal consilience has already shown that, due to their close mathematical relationship, the sufficiency and necessity over a set of two transitions behaves similarly to their information-theoretic generations, determinism and degeneracy, in conditions of increasing uncertainty [1]. Here, their similarity is shown with a larger set of transitions.

Specifically, a system composed of 8 states with self-loops of $p = 1$ was specified (see Fig.S1, top left). In order to vary the uncertainty about causes and effects, noise (uncertainty about effects) and common causes (uncertainty about causes), were introduced along two separate axes. The first axis increased uncertainty about effects by shifting the system down to a condition of complete randomness in terms of its transitions (an all-to-all Markov chain wherein all transitions $= 1/n$, which means the system behaves as unpredictably as possible). The second axis, uncertainty about causes, moved the system to the condition wherein all transitions had an identical set of effects (thus increasing the number of common causes).

For every step along the axis that increased the noise, the probability from each self-loop was redistributed equally across the other states in the system, with the total amount of probability redistributed being $1/steps$ each step. For every step along the axis that increased the number of common causes, the full set of transitions for a state (a row in the TPM) were replaced one at a time with a duplication of the first row until all distributions were the same. Changing the model along just this latter axis began with all states having unique state-transitions and ended with all states transitioning to a single state (see Fig.S1, bottom left). Finally, these two axes of changes to the system were combined such that at every step, both more noise in effects was introduced, and at the same step, more common causes were introduced (see Fig.S1, middle diagonal).

At each step, the system-wide sufficiency plus the necessity was calculated for each state, as well as the determinism plus the specificity, along the increasing uncertainty in effects axis (Fig.S2A), along the increasing common causes axis (Fig.S2B), as well as along both axes combined (Fig.S2C). As in the main text, this is done in a way to ensure the same $[0, 1]$ bounds (See Section 3.2).

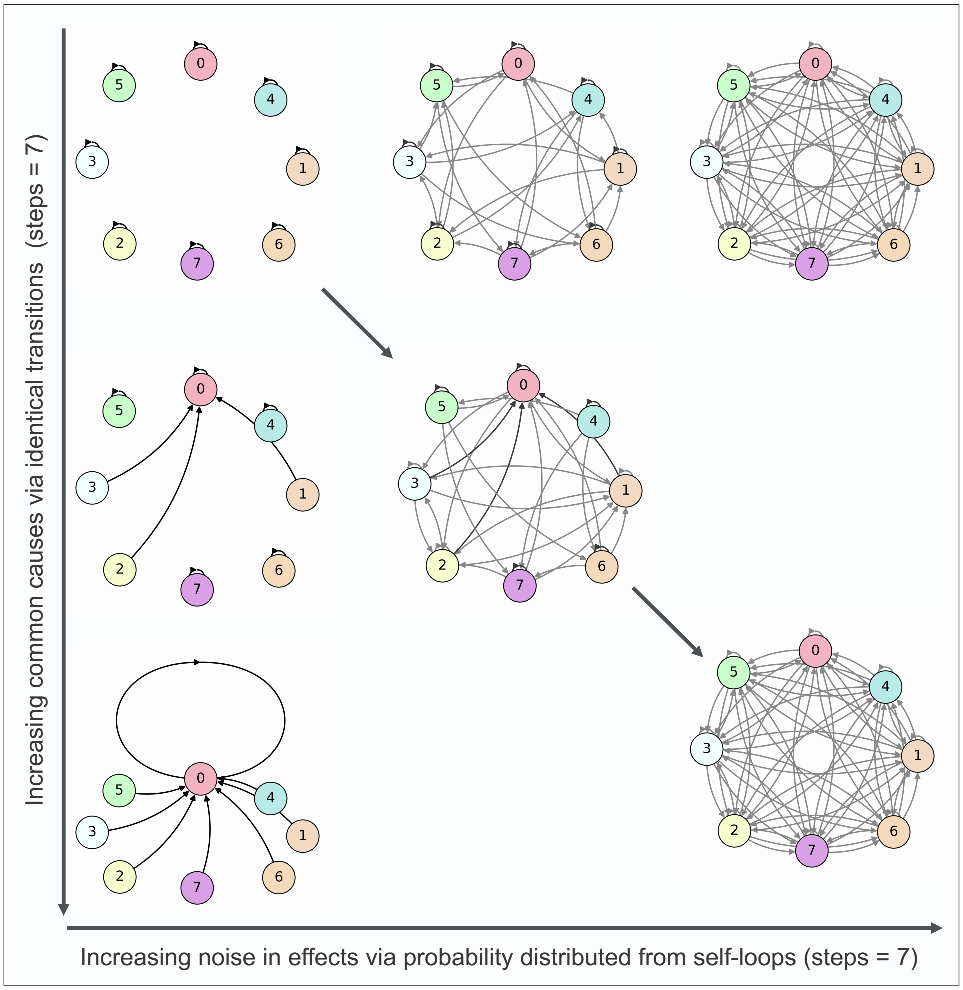

Figure S1: **Increasing uncertainty in the causal relationships of an 8-state system**. Starting in a state of self-loops with $p = 1$, states in the network were changed over a set number of steps equal to the size (number of states) of the system in three ways. Along the x-axis, self-loop probabilities were reduced by $1/steps$ and distributed equally to the other states (thus increasing the uncertainty of a particular effect, given a cause) until the system was an all-to-all network with random transitions. Along the y-axis, at each step a state was replaced with the transition distributions of another state (increasing the number of common causes and thus increasing the uncertainty of a cause, given an effect), until all states in the system shared the same transition. The system was also subjected to both changes at each step (the middle diagonal), ending again in an all-to-all state of random transitions.

Notably, all CP values changed similarly along both axes of steps that increased uncertainty about causes and effects, even in larger systems (values for 100 states and 100 steps are also shown in Fig. 2D).

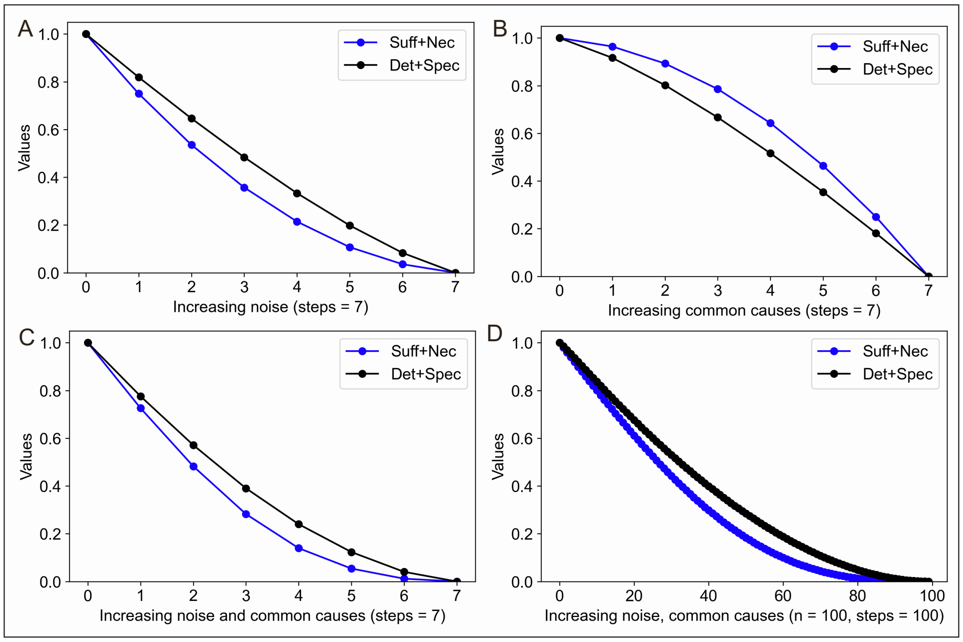

Figure S2: **Causal primitives vary together with uncertainty.** (A) The sufficiency plus necessity value and the determinism plus specificity value are shown to behave similarly across increasing uncertainty over effects (noise) as the probabilities of self-loops in the system (visualized in Figure S1) are redistributed across the system in increasing steps. (B) Similar behavior in response to increases in common causes (overlap). (C) Similar behavior in response to increases in both noise and overlap. (D) The measures still behave similarly as the system becomes larger and more steps are added.

S2 Calculating dynamical consistency across scales.

Not all macroscales are sensible summaries of their underlying microscale; indeed, some macroscales may be dynamically inconsistent when defined [2]. Much as in Klein and Hoel [3], here I deem a macroscale valid if it is *consistent* with its underlying microscale, with consistency being defined as whether the path of random walkers on the Markov chain is the same at

both the microscale and the macroscale (i.e., whether or not the macroscale acts as an accurate summary statistic for the microscale's dynamics).

Specifically, for a Markov chain the inconsistency can be defined as the Kullback-Leibler divergence [4], taken between an expected distribution of random walkers, across timesteps $t \to t_n$, in S vs. S_M, given an identical starting state on each scale. The result of this Kullback-Leibler divergence puts a numeric value on the information about dynamics lost at a macroscale, which can be quite small, and which can be minimized with more complex and inventive types of macroscales, like higher-order macroscales, instead of the kind of simpler coarse-grains used herein [3].

Indeed, in real-world systems, it may be preferable (or even necessary) to instead specify some threshold where small amounts of information loss about dynamics do not lead to a macroscale being discarded. However, here I enforce a strict notion of consistency, wherein a random walker is dropped at every possible state, and inconsistency between the macroscale and the microscale is summed over all of their moves for the next 5 timesteps. Any non-zero values imply inconsistent macroscales, which are discarded. Therefore, all macroscales considered herein are fully consistent with the dynamics of their underlying microscales.

S3 Pseudocode of CE 2.0's calculation.

What follows is a high-level description of applying the CE 2.0 analysis to a model system like those herein. It is modeled after the pseudocode used to help understand how to calculate similarly complicated theories like Integrated Information Theory [5].

Generally, given a microscale TPM, CE 2.0 mainly involves the identification of a set of possible partitions, the creation of macroscale TPMs via coarse-graining the microscale TPM based on those partitions, and the identification of a path respecting those partitions from the microscale to a macroscale with a TPM that possesses a maximal gain in the causal primitives. While each step is addressed in more detail in separate sections of the paper, the goal below is to provide a plain language overview.

1. **Given a starting TPM, treat it as the microscale, and calculate its causal primitives (CP)**. See main text, Section 2.2, for the details of CP's calculation (specifically, for these models herein, CP is equal to the determinism plus specificity).

2. **Derive the full set of possible partitions of the TPM's states**.

E.g., for a microscale with states $(0), (1), (2)$ the full set of possible partitions would be $(0, 1), (2)$; $(0, 2), (1)$; $(0), (1, 2)$; $(0, 1, 2)$.

3. **For each partition in the set of possible partitions, coarse-grain the microscale TPM, creating a macroscale TPM for each partition**. In the simplest case, as here, simply coarse-grain together inputs and outputs (for full details of coarse-graining, see Figure 4 of Klein and Hoel (2020) [3]).

4. **Test for dynamical consistency.**

    - For each macroscale TPM, calculate the dynamical consistency (see S2 of the SI for details).
    - If dynamical inconsistency > 0, then discard that TPM from the set of TPMs and the associated partition from the set of partitions.

5. **For the remaining TPMs, calculate their CP.**

    - Discard TPMs (and the associated partitions) for which the summed causal primitives are lower or equal to the microscale.

    At this point, what remains are the set of macroscales (TPMs resulting from coarse-graining based on a partition) that have a positive gain in CP against the microscale, and are dynamically consistent.

6. **Identify a path between microscale and macroscale.** Here, the chosen path leads from the microscale to the macroscale with the highest dimensionality and the highest gain in CP.

    - Order the macroscales by CP. If multiple macroscales are tied, then order them by path length. The winner is the endpoint.
    - For each path from the microscale to the endpoint, order them by length.
    - Take the longest path. If multiple paths are tied, pick one randomly.

7. **Calculate the differences in CP, (ΔCP), for each partition along that path**. The total non-redundant gain in CP is the total amount of causal emergence in the system, and the amount or degree of causal emergence is found by computing the difference between the endpoint's CP and the CP of the microscale.

8. **Normalize all the differences along a path as if they were a probability distribution.** This results in the causal contribution distributions, as shown in the main text of Figure 3, C and F.

9. **Compute the emergent complexity.** This is done by computing the entropy of the causal contribution distribution, as detailed in Section 4 of the main text.

S4 Heuristics for the CE 2.0 framework via the SVD

Zhang et al. [6] proposed a "vague" causal emergence calculation based on applying the singular value decomposition (SVD) to Markov chains. This is based in the average of the resultant singular values (σs), which they call γ, and which they prove reflects the average dynamical reversibility across system states. It was also shown capable of approximating the determinism plus specificity used herein (for details, see [6]). As they noted, this indicates a connection between the causal primitives and dynamical reversibility.

Furthermore, their research provided a way to approximate (quite precisely) the degree of causal emergence in the CE 1.0 framework (analogous to finding the macroscale with maximal EI). Specifically, they first identify a set of non-zero σ values, given some threshold, ϵ, and average these together ($\bar{\sigma}$). The difference between γ (the un-thresholded average) and $\bar{\sigma}$ is taken. When ϵ is a small non-zero value, and so includes most non-zero σ values, this method can elegantly track the maximal increase available in EI at some possible macroscale, without have to search across the set of scales, and so offers a precise heuristic for the degree of causal emergence in the CE 1.0 framework without any combinatorial explosions (only requiring the TPM of the microscale).

Here I show steps for how the SVD method can be adapted for the CE 2.0 framework (in which case, "vague" causal emergence becomes a specific value). To do this, the set of singular values can be used as proxies for *directionalities* of coarse-graining. The causal contributions of each directionality (represented by each σ) can then be calculated via an adaptation of the causal apportioning schemes detailed in Section 3.2.

Here, unlike in [6], the initial trivial σ_1 value is discarded, since it is always 1 or greater for any Markov chain and therefore reflects nothing about the causation of the system (assuming a set of σ values ordered by descending values). The average of the remaining values, here called $\gamma*$,

closer approximates the causal primitives of a system. This can be seen via the same simulations previously detailed in S1 of manipulating a system along increasing axes of uncertainty in its effects (via noise), as well as uncertainty in its causes (via common causes). For the same manipulation in the same system as S1, Fig. S4A plots the original state-averaged dynamical reversibility γ from [6] against the new $\gamma*$, and shows how they behave compared to the causal primitives. $\gamma*$ behaves very similarly to the determinism plus specificity (including, e.g., becoming zero when the system is at full randomness, while γ does not).

These adaptations put the SVD method more in line with the CE 2.0 framework. In this adaptation of the SVD method for CE 2.0, the total amount of causal emergence can be estimated as the highest-available gain that is non-trivial: $\sigma_2 - \gamma*$. When calculated in the "block model" system as in Figure. 4 of the main text, across the same scheme of probability redistribution, this value behaves similarly to the total gain in CP (plotted in Fig. S4B). In fact, in the initial configuration of the system prior to probability redistribution, the total gain of CP (in terms of sufficiency plus necessity) at the macroscale is actually identical to the $\sigma_2 - \gamma*$ value.

For comparison, the values applying the "vague" causal emergence from [6] are shown. Since that method approximates the gains in EI, it inherits some of the same limitations of the CE 1.0 framework. For instance, during probability redistribution of this type within a macroscale, the measure is mathematically unstable, reducing to zero following any probability redistribution of this kind within a macrostate (see Figure S4B for a plotted value, where using a low ϵ to include most non-zero σ values is labeled "CE 1.0 (SVD)").

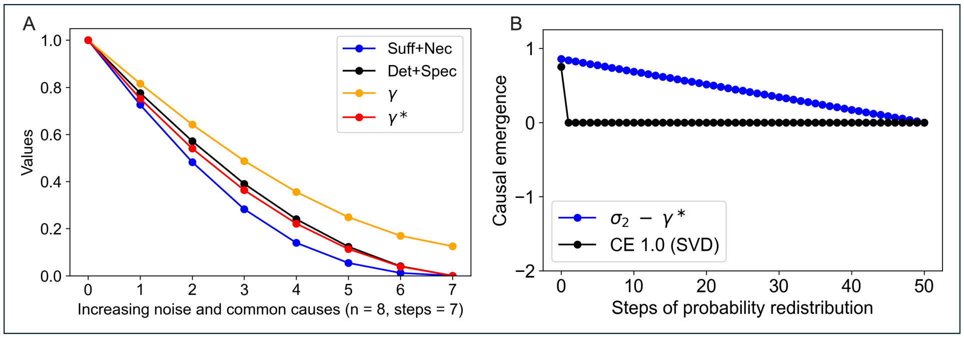

Figure S3: **Adapting the SVD method to CE 2.0 is demonstrated across two different models of probability redistribution** (A) For the same steps of increasing noise and common causes in the same system as in S1, the CP values are again plotted, but also the proposed changes to the averaged dynamical reversibility, wherein $\gamma*$ is used instead of $\gamma*$. (B) Plotting the measures for a different case probability redistribution, this time the same as in Figure 4 of the main text. There, a "block model" consisting of two equivalency classes has its probabilities redistributed until it is entirely composed of self-loops. Plotted in black are the values when using the dynamical reversibility method detailed in [6] to calculate causal emergence during this probability redistribution. Just as when using the EI, the measure detects no macroscale causation in most configurations. However, when using the adaptation of the SVD method for CE 2.0 proposed herein, the total gain $(\sigma_2 - \gamma*)$ operates similarly to the total gain in the causal primitives (compare to Fig.4 in the main text).

Furthermore, applying a version of the causal apportioning schema enables an SVD-based assessment of multiscale causal structure. Specifically, each σ (excluding σ_1) can be compared to the average of the remaining values, $\gamma*$. Causal contributions can be assessed for all σ_i (where $2 \leq i \leq n$) that are positive, and thus satisfy:

$$\sigma_i > \frac{1}{n-1} \sum_{j=2}^{n} \sigma_j$$

This way of using the SVD to identify the unique causal contributions of different scales was tested on the multiscale structure of the model systems used in Section 4 of the main text. For the first system in Figure 3 of the main text (Fig.3A-C), which lacks multiscale structure, this method results in just a single positive value from the multiscale SVD analysis:

0.61, indicating a single top-heavy macroscale (agreeing with the path-based analysis from the main text). Meanwhile, the second system (Fig.3D-F), which does have mesoscale structure, has three positive values: 0.489, 0.06, 0.06, indicating the presence of one or more mesoscales.

Ultimately, these results indicates that adapting the SVD method designed for the CE 1.0 framework in [6] is a promising heuristic for the CE 2.0 analysis.

References

[1] Comolatti, R., and Hoel, E. (2025). Consilience in causation: Causal emergence is found across measures of causation. Entropy *27*, 825.

[2] Rubenstein, P. K., Weichwald, S., Bongers, S., Mooij, J. M., Janzing, D., Grosse-Wentrup, M., and Schölkopf, B. (2017). Causal consistency of structural equation models. Preprint at arXiv arXiv:1707.00819.

[3] Klein, B., and Hoel, E. (2020). The emergence of informative higher scales in complex networks. Complexity *2020*, 8932526.

[4] Cover, T. M. (1999). Elements of information theory (John Wiley & Sons).

[5] Gomez, J. D., Mayner, W. G., Beheler-Amass, M., Tononi, G., and Albantakis, L. (2020). Computing integrated information (Φ) in discrete dynamical systems with multi-valued elements. Entropy *23*, 6.

[6] Zhang, J., Tao, R., Leong, K. H., Yang, M., and Yuan, B. (2025). Dynamical reversibility and a new theory of causal emergence based on SVD. npj Complexity *2*, 3.
